# The Thermodynamic Consequences of Parkinson's Disease

**DOI:** 10.3389/fneur.2021.685314

**Published:** 2021-08-26

**Authors:** Peter A. Kempster, Laura Perju-Dumbrava

**Affiliations:** ^1^Neurosciences Department, Monash Medical Centre, Clayton, VIC, Australia; ^2^Department of Medicine, School of Clinical Sciences, Monash University, Clayton, VIC, Australia

**Keywords:** Parkinson's disease, energy, dopamine, weight loss, thermodynamics

## Abstract

Several lines of evidence point to a pervasive disturbance of energy balance in Parkinson's disease (PD). Weight loss, common and multifactorial, is the most observable sign of this. Bradykinesia may be best understood as an underinvestment of energy in voluntary movement. This accords with rodent experiments that emphasise the importance of dopamine in allocating motor energy expenditure. Oxygen consumption studies in PD suggest that, when activities are standardised for work performed, these inappropriate energy thrift settings are actually wasteful. That the dopaminergic deficit of PD creates a problem with energy efficiency highlights the role played by the basal ganglia, and by dopamine, in thermodynamic governance. This involves more than balancing energy, since living things maintain their internal order by controlling transformations of energy, resisting probabilistic trends to more random states. This review will also look at recent research in PD on the analysis of entropy—an information theory metric of predictability in a message—in recordings from the basal ganglia. Close relationships between energy and information converge around the concept of entropy. This is especially relevant to the motor system, which regulates energy exchange with the outside world through its flow of information. The malignant syndrome in PD, a counterpart of neuroleptic malignant syndrome, demonstrates how much thermodynamic disruption can result from breakdown of motor signalling in an extreme hypodopaminergic state. The macroenergetic disturbances of PD are consistent with a unifying hypothesis of dopamine's neurotransmitter actions—to adapt energy expenditure to prevailing economic circumstances.

## Introduction

The prevalence of weight loss as a symptom suggests that many patients deviate from normal energy balance as Parkinson's disease (PD) progresses (1). Weight is often lost in phases during its course, followed by periods of stabilisation. Occasionally, weight change is rapid and severe enough to prompt investigations for malignancy. Many patients with advanced disease have low body weight and depleted fat stores. Alterations on the energy supply side of the equation are easier to identify. Depression, cognitive impairment, and olfactory deficits diminish appetite. Chewing and swallowing are impaired in advanced PD. Altered gut motility may affect absorption of nutrients.

PD patients have involuntary motor disorders that consume muscular energy—resting tremor, static muscle activation in rigidity, drug-induced dyskinesia and dystonia. Many PD patients are less physically active than healthy individuals. There is, however, evidence that the chief parkinsonian motor sign of bradykinesia involves defective energy of movement. That the dopaminergic deficit of PD creates a problem with energy efficiency highlights the role played by the basal ganglia, and by dopamine, in thermodynamic governance.

Thermodynamics is the study of energy in transformation. Its special term entropy is not easy to define—it means dispersed or unusable energy, randomness as opposed to order. The thermal energy of a cup of coffee becomes less concentrated as it spreads to its surrounds on cooling. The cup tips over, potential energy becomes kinetic energy as the liquid splashes on the floor, coffee molecules no longer confined to a vessel but distributed randomly all about. One does not observe such processes running in reverse. Energy is conserved when it transforms, but things adopt their most probable dispositions as it does so. There is, seemingly, an exception to these thermodynamic principles—living systems, which maintain wildly improbable degrees of complexity. Organisms achieve internal order through interactions with their environments. These are the iron laws of the physical world and even conditional suspensions turn out to be temporary. Entropy resumes its relentless march with death and decomposition.

While living systems are complex, they don't directly monitor or control their complexity. What they control is energy. Molecular mechanisms achieve this at a cellular level. For animals, macroenergetics rely heavily on the nervous system. Motor programming enables energy acquisition and manages the energy usage of skeletal muscle. This article will review the thermodynamic effects of PD. In addition to changes in energy balance, we will be looking at the role of signalling within the motor system. The flow of energy in the human body is regulated by the smaller amounts of energy that are used for the flow of information. Energy and information have a deep relationship, which converges around the concept of entropy (2).

## Energy and Dopamine

Virtually all animals share certain attributes—ability to move, reliance on chemical energy ultimately sourced from plants, a central control network. While survival and reproductive fitness drive the Darwinian model of evolution, an animal's ability to capture energy is a vital pre-condition for success. Adaptive behaviours, whatever their goal, require energy efficiency in execution.

Dopamine's fundamental role may be as a token of energy. The idea of dopamine as the neurochemical of reward is well-accepted. Human impulse control disorders, resulting from direct or indirect overstimulation of dopamine receptors, emphasise hedonistic and addictive behaviours (3). Mesolimbic and mesocortical projections are the dopaminergic inputs to this “reward system,” which integrates cognitive, emotional and motor planning resources into complex behavioural responses.

Evidence from rodent experiments points to motor energy as the link between dopamine and food reward (4). Blocking or depleting dopamine in rats reduces the amount of physical but not cognitive effort that the animals will devote to obtaining food (5). The effects do not resemble those of appetite suppression by pre-feeding or by drugs, and hedonistic reactivity to sucrose is not sensitive to dopamine blockade (6, 7). Mice rendered hyperdopaminergic by knockdown of the dopamine transporter gene expend more energy to gain food (8). If availability is not constrained, this effort does not translate into an overall increase in consumption (they consume larger, less frequent meals). These experiments can be interpreted as showing that increased dopamine made the animals undervalue the energy outlaid on reward-seeking activities—the nexus between effort and reward gained was weaker, not stronger. But when food is harder to obtain, normal homeostatic control occurs, suppressing activities with unfavourable cost-benefit ratios (9). As will be discussed below, simple movements seem also to show an energy thrift effect, inappropriately prominent in PD and resulting in a loss of “vigor.”

Energy-marginal environments have pertained to much of human evolution (10). The progressive encephalization in humans and other primates involved energy trade-offs with gut size (11). Late life energy stresses associated with the human trait of longevity may predispose to neurodegeneration. Dopamine, in regulating movement and reward-seeking, has been integral across species to balancing energy outlay and input. Its functions are strongly conserved by evolution. Lamprey, jawless fish of ancient lineage, have, of all vertebrates, the most distant phylogenetic relationship with humans. The last common ancestor lived 560 million years ago. Regulating the restricted repertoire of lamprey motor activity, dopamine modifies striatal output with the same general pattern as mammals—a direct pathway with neurons expressing D1 receptors, and a D2 indirect pathway (12).

## Energy Balance in PD

Some energy imbalance in PD can be traced to its motor deficits, which show a strong relationship with nigral cell loss and the resultant dopaminergic deficiency (13). The neuropathology of PD, though, affects other neuronal populations and other neurotransmitter systems. This contributes to the manifold reasons for reduced body mass in PD.

### Weight Loss

Patients with PD have lower body weight in comparison with age-matched subjects, and are more frequently underweight (1, 14). A systematic review showed that between 0 and 24% of PD patients were malnourished, while a further 3–60% were at risk of malnutrition (15). Weight loss across the disease course is about 3–6 kg but can be as high as 12 kg (16). Malnutrition and unintended weight loss seem to correlate with disease progression (17), and are negatively associated with quality of life (18).

Low body mass index is correlated with lower dopamine transporter activity (19). There is an inverse relationship between body mass index changes and Unified Parkinson's Disease Rating Scale motor score (20). Moderate or severe dyskinesia is also a risk factor for undernutrition (21).

Non-motor symptoms have been linked with weight loss in PD patients (22). Some studies show association of low protein intake with a lower olfactory score (23, 24). Impaired gastric emptying and bacterial overgrowth from small intestine dysmotility could affect nutrition, though correlations with body mass index were weak in a small number of studies (25–27). PD patients with cognitive impairment or depression are at increased risk of weight loss and malnutrition, presumably because of effects on appetite and feeding (28–30). Dysphagia may contribute to weight loss (31).

The mesolimbic dopamine system is modulated by nutritionally significant hormones (32). Leptin, which decreases food intake, can change food desire. Leptin attenuates the response of brain reward regions to food stimuli, and enhances activation of regions involved in cognitive inhibition. Ghrelin, on the other hand, is a hormone that stimulates food intake by enhancing the hedonic and incentive responses to food-related cues. Leptin and ghrelin levels were lower in PD patients who had lost weight (33).

In a prospective study, PD patients began to lose weight loss shortly before the diagnosis (17). They tended to increase their energy intake as their weight fell. Functional neurosurgery is associated with weight gain, and this effect is stronger for procedures that target the subthalamic nucleus (STN) (34). There may be a correlation with reduced dyskinetic movement (35, 36). After deep brain stimulation (DBS), weight gain appears to occur without significant increase in appetite or food intake (37).

### Resting Energy Expenditure

**Figure 1:**
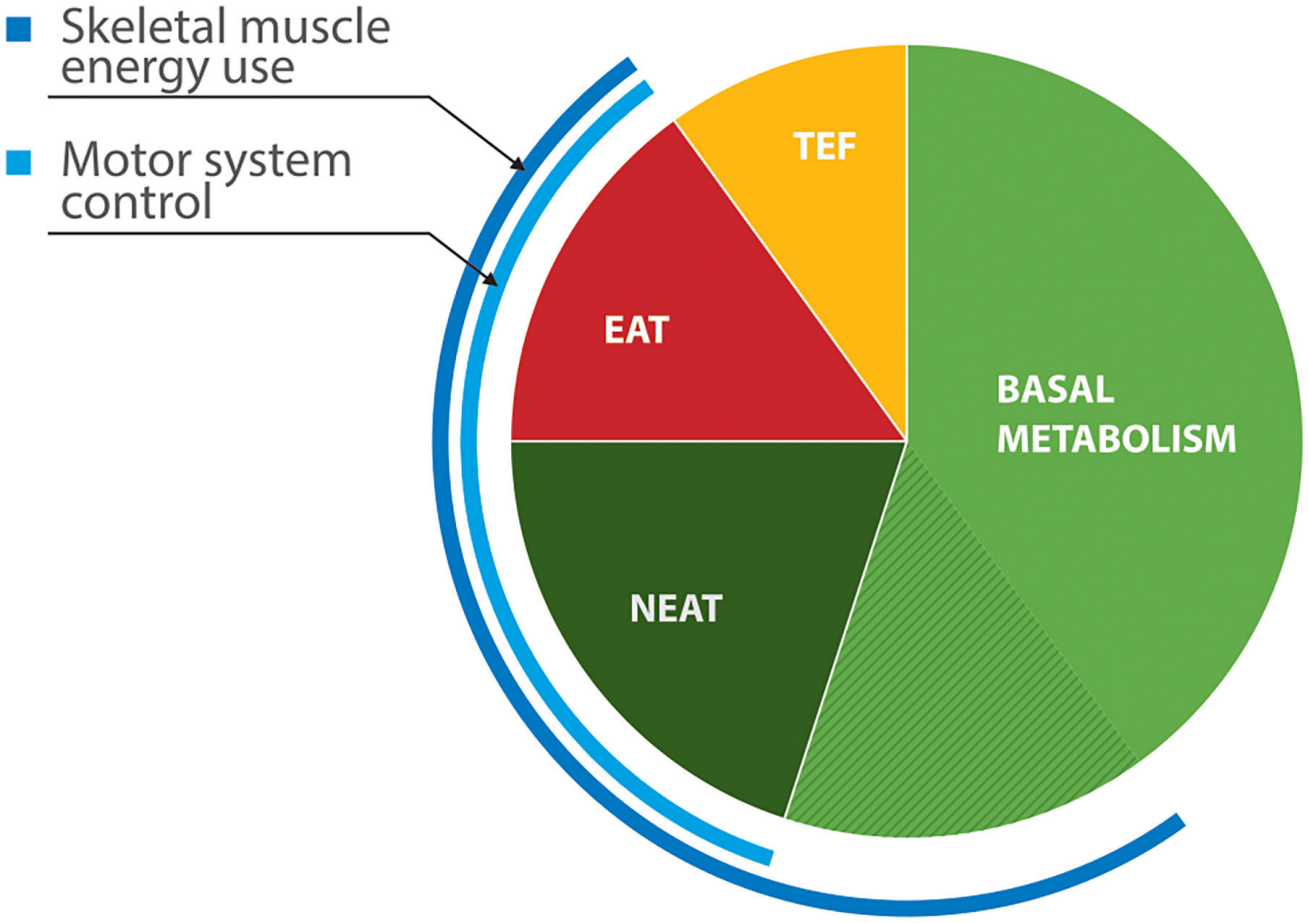
Components of energy expenditure in non-obese healthy adults (39, 40). EAT, exercise activity thermogenesis; NEAT, non-exercise activity thermogenesis (low-level physical activities of daily living, including some locomotion, upper limb movement, postural tone and maintenance); TEF—thermal effects of food (digestion and secondary metabolism). Skeletal muscle contributes roughly 20% of basal metabolic activity.

Resting energy expenditure (REE), the energy output in the absence of physical activity, represents about 60% of daily energy output (38). While some of this is basal metabolic activity, contributions from skeletal muscle to total expenditure are considerable (Figure 1). Involuntary motor features of PD—tremor, rigidity, and drug-induced dyskinesia—could elevate energy consumption at rest. Evidence of abnormal REE in PD is, however, inconsistent. Indirect calorimetry estimates metabolic energy use from concentrations of oxygen and carbon dioxide in exhaled air. In studies that have shown increased REE, (41, 42) rigidity rather than tremor correlated better with REE measured in *off* states. Capecci et al. (43) found that REE fell by 8% after doses of dopaminergic medication, although subjects in another study who developed active *on* phase dyskinesia had a rise in their REE (41). Other researchers have found normal REE in PD, even in patients who had been losing weight (44, 45).

Reduction in tremor, rigidity and dyskinesia after DBS surgery could all have contributed to the reduced REE shown in several studies (35, 36, 46). Another, though, reported no change in REE after STN-DBS (47).

### Physical Activity and Energy

While physical activity is probably reduced overall in PD (44), comparisons of exercise at standard workloads point to an inefficiency of movement-related energy expenditure. Across various speeds of treadmill walking, the oxygen consumption rate of parkinsonian subjects exceeded that of normal controls by a 6–10% margin (48). Oxygen consumption at rest was no different in this study, suggesting that reduced walking economy in PD relates to physical activity and not to tremor or rigidity. Kalifa et al. (49) compared early PD with control subjects on matched cycling at moderate intensity and also showed increased oxygen consumption with exercise. In another cycling study, parkinsonian patients reached similar maximum oxygen consumption to controls, but at lower power outputs, again consistent with reduced economy of movement (50).

### Bradykinesia and Energy of Movement

Bradykinesia is a shorthand for complex disturbances of initiation and execution of actions and the ability to sustain them (51). Akinesia (failure of initiation) and hypokinesia (underactive movement) both relate to bradykinesia, as does the sequence effect—repetitive movements becoming smaller or slower. Slowness itself is not always present, as is seen in the phenomenon of festination—a gait that hastens by rapid, small steps—or spoken words that run together at speed and are hard to understand. Loss of motor energy may be its defining character.

Fast goal-directed limb movements are executed by triphasic bursts of muscle activation in an agonist-antagonist-agonist sequence (52). In PD, this basic motor architecture is intact, but burst size is inadequate to impel the limb to its destination (53). Additional burst cycles need to be recruited, and the movement is deficient in acceleration and peak velocity. Movements are always underscaled in relation to their intended speed and range, though not necessarily in absolute terms (54). Thus, a large movement might comprise bursts that would have been adequate for a normal smaller one.

Parkinsonian movement occurs over an abnormally narrow dynamic range, consistent with reduced “motor motivation” (55). Rapid movements made by healthy subjects can be shown to be influenced by both speed-accuracy and energy cost trade-offs. PD patients balance speed and accuracy normally but assign a higher energetic cost to movement (56). The energy expense of a motor command corresponds to the force generated by the encoded action. In upper limb force-matching tasks, PD patients underestimate the force generated by the contralateral hand, experiencing the same subjective sense of effort at lower energy output than controls (57). Using a force-based definition of motor energy, Tinaz et al. (58) demonstrated that the sequence effect of bradykinesia can be explained as an inability to meet the cumulative energetic demand of a repetitive task. Simultaneous dual tasks seem to magnify the problem of deficient motor energy, with PD patients struggling sufficiently to energise a second task (59). Higher motor planning is affected—PD patients cannot augment motor effort to track unpredictable targets in a choice reaction time task (60). It has been hypothesised that changes in electroencephalographic beta oscillation power in relation to movement is a cerebral cortical manifestation of abnormal energy regulation in PD (61).

In PD, therefore, patterns of voluntary muscle activation are consistent with a scaling back of motor commands, as if to conserve energy based on inappropriately high estimations of energetic cost. More normal scaling can be temporarily restored by additional attentional resources mediated by sensory cues (62). Movement speed in PD is responsive to behavioural motivation, as it is in healthy subjects. In PD, reward had a weaker effect on energising movement than an aversive stimulus (63), recalling the kinesia paradoxica effect first reported in the pre-levodopa era (64). Avoidance is the opposite of reward-seeking, although both are subject to a cost-benefit calculation about how much energy should be invested in an action.

The usual movements of parkinsonian subjects look slow and under-powered. Yet recruitment of additional muscle contraction is required to complete a hypokinetic action (53), and underinvestment of energy may in the end make it more costly to move. Oxygen consumption research previously cited is consistent with a degree of inefficiency and wastefulness.

### Cellular Energy Considerations

Abnormalities of energy metabolism, particularly mitochondrial function (65), have been theorised in the aetiology of PD. Certain mitochondrial defects could also increase the basal metabolic component of REE in PD by causing a degree of uncoupling of oxidative phosphorylation, and there is some evidence that brain heat production is increased in PD (66). Neuropathological research alludes to possible energy influences in selective neurodegeneration. As Braak pointed out, the neurons that are susceptible to α-synuclein deposition share certain characteristics (67). All are projection neurons that have axons disproportionately long and thin for their cell body size, and are unmyelinated or poorly myelinated (68, 69). A long, thinly myelinated nerve fibre requires more energy for impulse transmission than a shorter well-myelinated one (70, 71). For neurons of the substantia nigra, two additional factors are at play—an energy-expensive “pacemaker” function (72), and a high degree of axonal arborisation (73, 74). A broader discussion about oxidative stress (75) and other molecular mechanisms for nerve cell degeneration in PD that concern energy production (76) is, however, beyond the scope of this review.

## Entropy and the Motor System

Living things exist because they can restrict themselves to a narrow range of improbable states. They do this by acquiring energy in an organised, usable form—free energy—then by controlling its transition to an unavailable form, the random molecular motion of heat. In animals, movement occupies a central place in this process.

### Thermodynamic Entropy

Entropy means disorder, medical students are taught in their foundation scientific studies. Thermodynamics started with the Industrial Revolution and enquiries into the conversion of the energy of steam into mechanical work by cyclical engines. Ludwig Boltzmann (1844–1906), the Austrian physicist who gave entropy its commonly used mathematical expression, considered the degree of statistical randomness of molecules in an enclosed gas. This notional quantity defines the entropy of the system. If all particles are moving with complete unpredictability, the entropy is maximal. It is much more likely for gas molecules to behave this way than, say, all line up on one side of the container. The second law of thermodynamics states that entropy does not decrease in a closed system. The universe tends to an ever-greater state of disorder, the probability of this process corresponding to the arrow of time. Biological systems maintain their order while increasing entropy in their surroundings by a greater degree.

**Figure 2:**
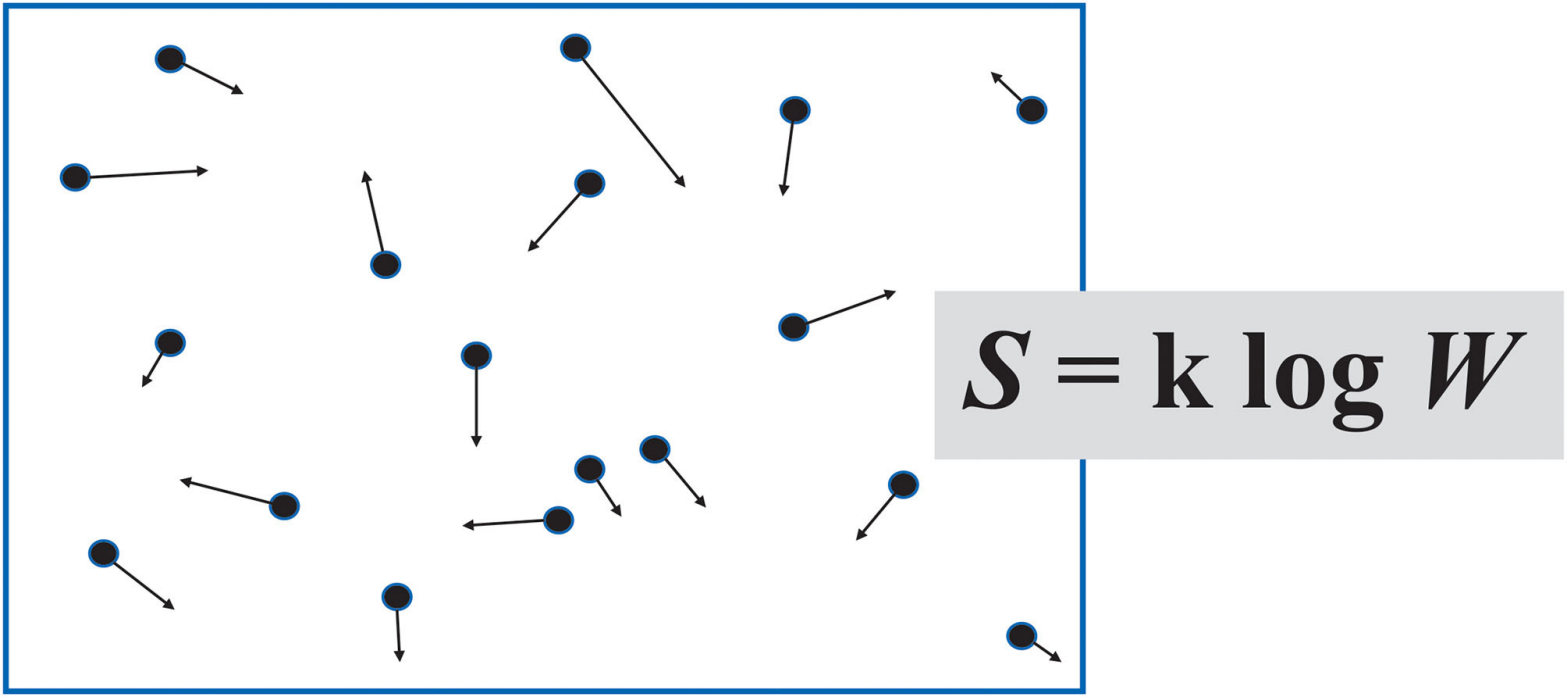
Boltzmann imagined a container of gas as a set of “microstates,” based on the position and momentum of its individual molecules. In his formula for entropy *S*, W represents the number of microstates, were it possible to count them.

Boltzmann's idea of a list of “information” about the particles of a gas (Figure 2), with the longest list corresponding to the most probable, most random, highest entropy macrostate, leads on to the use of the term in information theory.

### Information Entropy

At the end of World War 2, the mathematician Claude Shannon (1916–2001), who had worked on cryptography during the conflict, turned his attention to peacetime communication applications. His 1948 publication “A Mathematical Theory of Communication” (77), concerning coding and compression in the transmission of data, was a starting point of the discipline of information theory. Recognising the essentially probabilistic character of “entropy,” he fashioned a usage that disconnected the term from the physical world—the degree of randomness or unpredictability in a stream of symbols that compose a message. Shannon's entropy determines the flow of *information*, his term henceforth italicised, for it carries another shade of meaning from its “facts provided or learned” dictionary definition.

**Figure 3:**
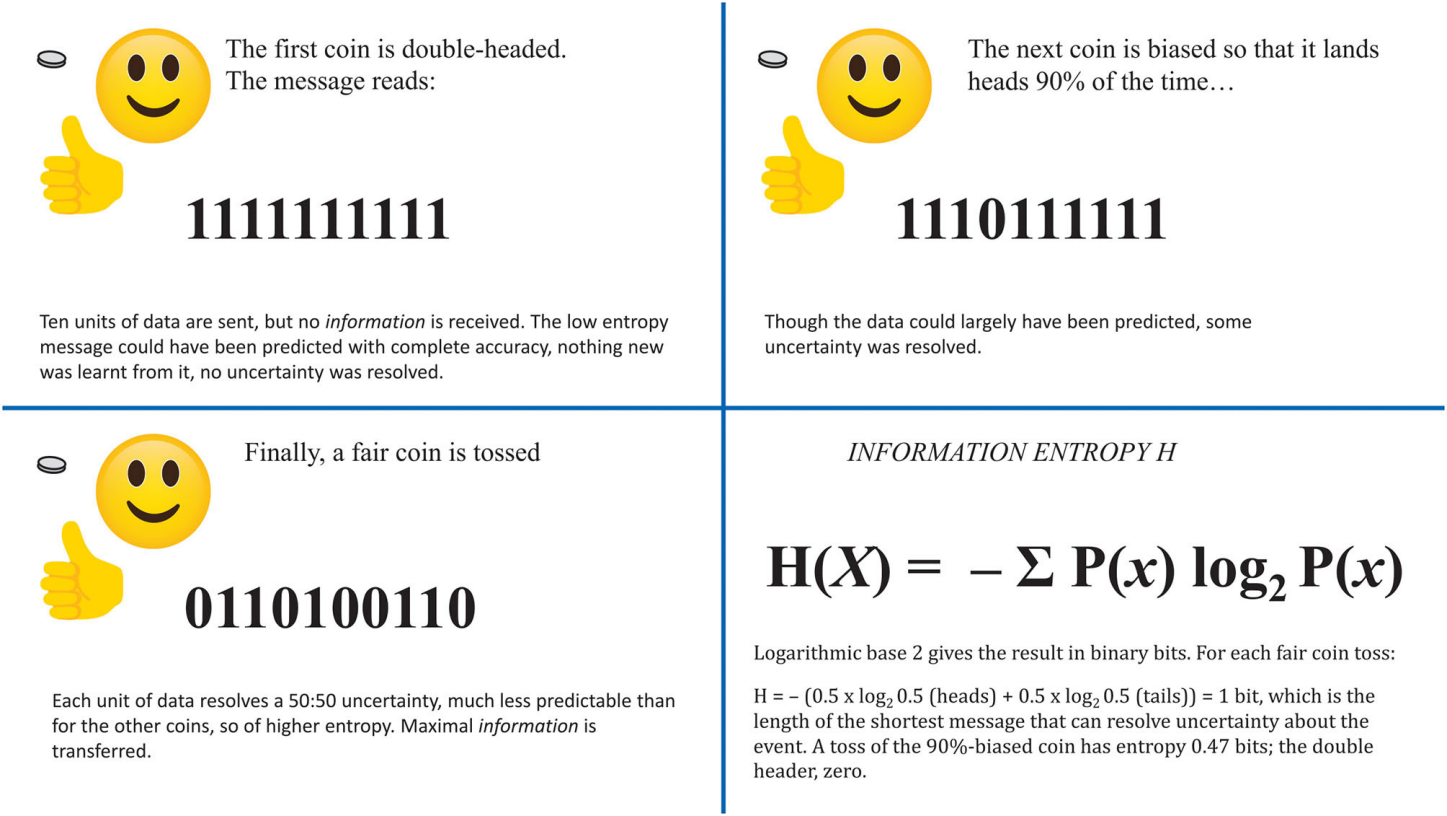
Three coins have known but different probability profiles. Each is to be tossed 10 times. A sender wants to transmit the results in a binary stream, 1 = heads, 0 = tails. Bottom right: a general expression for information entropy *H* takes the probabilities *P*(*x*) of the outcomes of a variable *X*.

As illustrated in Figure 3, *information* is the reduction of uncertainty by transmission of data that could not easily be known or predicted. Synonymous with *information* in information theory is *surprise*—that which is not expected. A message is informative by the degree to which its content is surprising. The expression for information entropy has a resemblance to Boltzmann's equation.

Shannon's principles were developed for the intentional communication of meaningful messages. Entropy quantifies unpredictability and describes the performance of a channel for any and all messages that might be sent over it. *Information* refers to how much (not what) has been understood from an actual message. A truly random distribution of symbols, could, with appropriate coding, transmit the maximum amount of *information*, and could not losslessly be compressed. Entropy, though, is the theoretical capacity to send *information*; meaningless random noise has high entropy but does not inform because uncertainty about the vast number of possible messages it might contain cannot be reduced. Alphabetic English has lower entropy (E and T are common, X and Z rare, Q is nearly always followed by U, etc.); this degree of predictability places a limit on *information* transfer though it allows some compression (as in Morse code—E and T are sent by single keystrokes while Z needs 4).

### Energy and Information

Information theory is a branch of mathematics and thermodynamics describes properties of the physical world. Yet information and energy have links that go well beyond some parallels in their mathematical formulae. Information needs to be registered, processed or erased by a physical system. This requires energy, usually electromagnetic, to be dispersed. It is possible to calculate rough comparisons between information gained (in bits) and energy transformed (in joules) for the transmission of digital message between two smartphones. It costs about 10,000 ATP molecules to send one bit of information across a synapse in the retina of a fly (79).

Communication of information requires energy and transformations of energy involve information. What distinguishes usable, or free, energy from the randomness of thermodynamic equilibrium is *information*—reduced uncertainty. Carbohydrate molecules possess *information* because it is known that their atoms are bound together in a certain way and not all moving randomly about. In this sense also, *information* is physical (80).

As will be discussed, a “free energy” characterisation of information underpins an ambitious theory about self-organisation in the nervous system.

### Entropy Measurements of Biological Data

**Figure 4:**
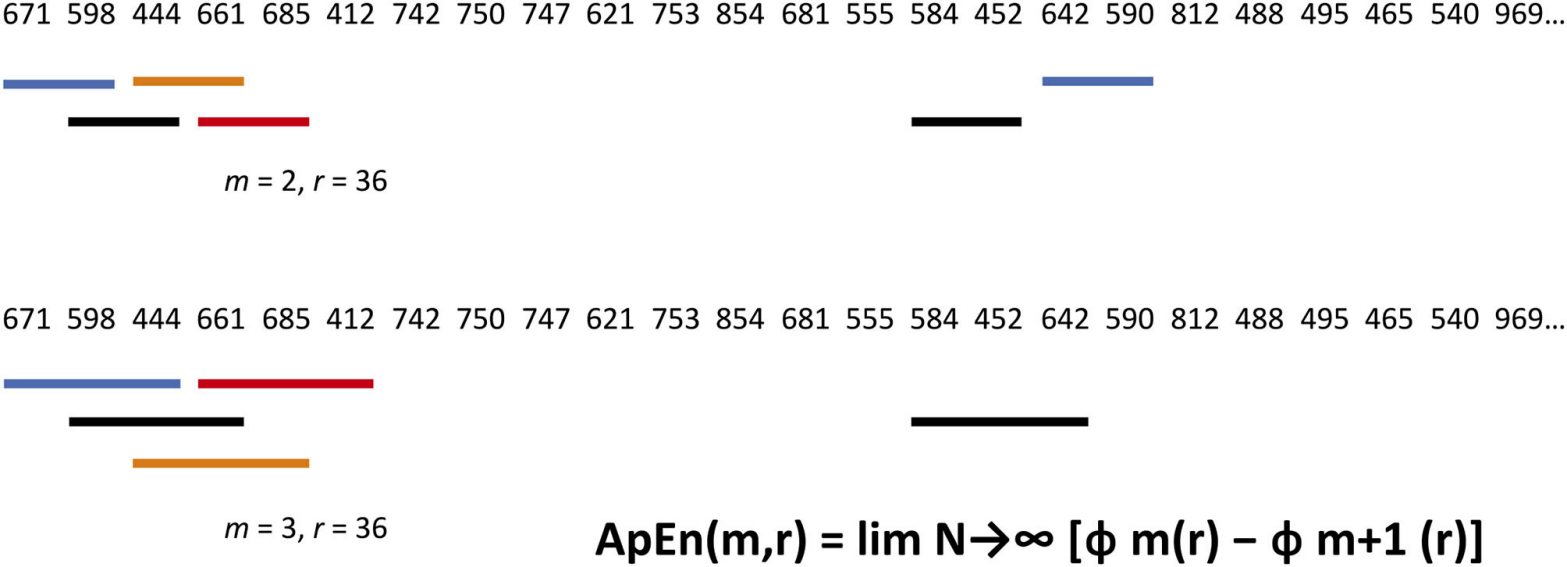
Approximate Entropy algorithm applied to an annual rainfall (mm) series for Melbourne. Statistical properties of marginal probabilities are a shortcut to the probability character of a whole sample. A block size *m* and scaling or filter parameter *r* are selected. Here, *m* = 2, and *r* = 25% of the standard deviation. Blocks are assessed in turn for matches—BOTH corresponding numbers must lie within the ± 36 filter. In the top row, Block 1 matches with Block 17, and Block 2 with Block 15. In the bottom row, *m* has been raised by one—all 3 numbers must correspond to within ± 36. Block 2 with Block 15 is a match. All *m* = 2 and 3 matches are then tallied to calculate Approximate Entropy. Interested readers can find theoretical and practical treatments of information entropy measurement elsewhere (78).

Biological data in a time series have dynamic irregularities that are not captured by “linear” time and frequency analysis. By considering the degree of randomness vs. predictability in a signal, Shannon's information entropy concepts can be used to infer additional layers of temporal organisation in nervous system activity. There are a number of entropy metrics, beginning with Pincus's 1991 work on approximate entropy (81). They quantify repetition as a marker of predictability by comparing short segments of data in a time series (Figure 4).

The question then arises as to what biological quality is being captured by entropy analysis. Entropy, because it gives additional capacity for coded information, is sometimes considered a measurement of complexity (82). The words, though, are not synonymous. A system that is simple and uniform is likely to have a regular, relatively predictable output of low information entropy. But a completely random system, with no attributes of biological complexity, would transmit a structureless, high entropy signal. True biological complexity sits at an intermediate point on this spectrum. Diseases of the motor system could cause alterations in both directions—loss of processing capacity resulting in simplified output (of low entropy) that imposes rigid, less adaptable control; or more random, higher entropy signals that reflect network disarray.

## Entropy Measurements in PD

Analysis of gait from wearable accelerometers during normal living shows increased entropy in PD (83). Accelerometer recordings of standing postural stability show the same trend—sample entropy in all three axes is significantly greater than for healthy older subjects (84).

Tremor, a predictably regular phenomenon in its own right, shows different entropy tendencies to gait and balance. Kinematic and electromyographic recordings of parkinsonian tremor have lower approximate entropy compared with physiological tremor in healthy controls (85, 86). Both STN-DBS and dopaminergic medication increase entropy of parkinsonian tremor (87, 88).

Recordings of neuronal activity in the basal ganglia show a fairly consistent pattern—the parkinsonian state has increased entropy. This is seen in interspike intervals, which presumably transmit coded data. Intra-operative recordings from the globus pallidus interna during DBS procedures show higher neuronal approximate entropy in PD compared with dystonic patients (89). In the MPTP primate model of PD, DBS of the STN reduces entropy in globus pallidus interna neuronal activity (90). During intra-operative recordings in PD patients undergoing DBS procedures, entropy in STN firing pattern went down when doses of subcutaneous apomorphine that ameliorated parkinsonism without inducing dyskinesia were administered (91). Recordings from freely ambulant parkinsonian patients implanted with STN-DBS showed, under medication-off and DBS-off conditions, greater entropy in those subject to gait freezing, especially when freezing was actually occurring (92). Electroencephalographic activity in PD shows increased entropy in comparison to healthy controls (93).

The concept of information entropy is independent of coding, content, and type of channel. What can be surmised in PD is that the configuration of the flow of information about energy of movement is altered.

Darbin et al. (94) have proposed an entropy hypothesis for neuronal firing in basal ganglia disorders. Low entropy neuronal activity could result in hyperlegible messaging and hyperkinetic motor output. On the other hand, high entropy signals, as observed in PD, give rise to hypokinesia. A high entropy channel could carry more coded information, or more random noise. But less predictable signals would likely require greater processing to decipher them. They would also be less compressible without loss of meaning, interfering with the way that motor transmissions must be funnelled from larger to smaller neuronal populations when descending the motor control hierarchy (95). High entropy in the parkinsonian basal ganglia appears to result in “pathological” information transfer, partly rectified by dopaminergic drugs or partly blocked by DBS (96).

## Predictive Coding of Movement and Free-Energy

The brain is a device that tests hypotheses about the external world. According to this theory, neural circuits employ a version of Bayes' rule for the conditional probability of events, whereby expectations—*predictive beliefs*, or *prior probabilities*—are updated in response to new evidence. This has become an important principle in understanding human perception, and in machine learning programmes that try to mimic it. Bayesian *perceptual inference* may approximate a neural coding principle underlying a continuous process of prediction error minimisation between forecast and actual sensory input that operates across all levels of the hierarchy of the central nervous system (97, 98).

Predictive processing encompasses the control of movement. Prediction error minimisation can also be achieved by changing sensation through action to make it fit with expectations—*active inference*. That is, the brain anticipates the proprioceptive outcome of a motor command. The theory posits a simple algorithm enabling the brain's complex hierarchical balance between “top down” projections of expectation and “bottom up” sensory traffic. While it is possible to show that perception and movement function as if Bayesian hypothesis-testing is embedded in the neural circuitry, it is not clear how such processing might have become established and evolved to its present level of sophistication.

Karl Friston, one of the originators of the predictive coding account of brain activity, has appealed to thermodynamics for an answer to this question (99). Living systems exist by restricting themselves to a small range of improbable states. By creating boundaries with the disordered outside world, and by exchanging energy and matter with it, they cause these to be the most probable internal arrangements. As Claude Shannon used “entropy” as a term for randomness in a data stream, so Friston links the prediction error of predictive coding with the thermodynamic variable of free energy. *Free-energy* (italicised to distinguish from its thermodynamic counterpart), he writes, “is an information theory quantity that bounds the evidence for a model of data” (99).

In thermodynamics, free energy is the energy available to do work. The relevant equation can be paraphrased;

*Free Energy Equals Total Energy Minus Entropy*

In performing mechanical or chemical work, some free energy is dissipated into entropy—the random molecular motion of heat—which, in a closed system, is unavailable to do work. In an inefficient engine, or an explosive chemical reaction, nearly all free energy might be so dispersed. Biochemical free energy, which drives the direction of reactions, is minimised when a protein folds to its functional 3-dimensional conformation. The probability of finding a molecular system in one state as opposed to another is determined by the difference in their free energies (100). It is therefore more probable that proteins occupy desirably folded steady states than undergo further thermodynamic transition. Living systems minimise free energy to maximise the chance that their complex, improbable elements will retain their stability.

An accurate model of an action yields predictable incoming sensory signals of low “surprise” content. Yet the brain has no innate yardstick for entropy, or surprise. Rather, it controls the “free energy” character of information encoded in probability distributions of expectation, which is minimised by reducing prediction error. If returning signals are unsurprising, probability distributions don't require updating to new data. Using complex mathematics, Friston shows that calculations based on *free-energy*, which places an upper limit on surprise, approximate Bayes' rule. *Free-energy*, abstract informational quantity, exists through actual neuronal energy, which is needed to code its probability estimates (80). Accurate predictions are frugal with thermodynamic free energy, since little additional neuronal activity is needed to modify these distributions to unsurprising feedback. Friston says that any self-organising system that is at nonequilibrium steady state with its environment must, behind physical and statistical boundaries, minimise its *free-energy* (101). By doing so, it will continue to occupy its small set of preferred states.

The *Free-energy* principle has been described as a piece of philosophical-mathematical reasoning that seeks to explain nervous and mental phenomena (102). At another level, it says that life depends on exploiting relationships between energy and information to achieve thermodynamic outcomes. This cursory and unmathematical synopsis of *Free-energy* shows what might be happening in sensori-motor neurotransmission, though the theory can only be applied conceptually to PD. Underscaled bradykinetic movements that have to be augmented by secondary muscle activation, or that decrement on repetition, imply that *free-energy* is not being minimised in PD. “Surprising” sensory feedback delivers continual prediction errors on actions that fall short of expectation.

## Malignant Syndromes of PD as Thermodynamic Crises

In its fully developed form, neuroleptic malignant syndrome is a life-threatening combination of pyrexia, rigidity with rhabdomyolysis, autonomic instability and altered mental state (103). There are milder versions, with a continuum that extends from severe drug-induced parkinsonism. Dopaminergic D2 receptor blockade by antipsychotic drugs is likely to be the primary cause, and peripheral explanations around skeletal muscle fibre toxicity are less satisfactory. This conclusion is reinforced by the occurrence of neuroleptic malignant-like states in PD (104–108). Sudden withdrawal of dopaminergic therapy, intercurrent infection and dehydration are the most common triggers. It can emerge from profound *off* phases in patients with motor fluctuations (109). Virtually all of the features of a neuroleptic drug-induced malignant state are reproduced in its PD counterpart (110).

Energy dysregulation is central to the malignant syndrome in PD. The metaphor of “meltdown” captures the runaway thermal effects. In a state of extreme parkinsonism, there little capacity for muscles to perform voluntary mechanical work. Instead, abnormal muscle contraction liberates large amounts of heat energy. Thermoregulation is overwhelmed. Some inbuilt load-limiting governor is overridden, muscles exceed their metabolic safety points, and start to break down.

Malignant syndromes underline the extent of the energy resources normally held in harness by the motor system. They bring into relief thermodynamic roles of dopamine in the basal ganglia by showing the consequences of these extreme hypodopaminergic states. Chemical energy stores in skeletal muscle are dissipated as entropy, while the complexity of muscle cells themselves is degraded by rhabdomyolysis. Information entropy of motor signals in malignant syndromes have not been studied, but some inferences can be made. Continuous involuntary skeletal muscle activation implies motor messages dominated by random output, far from the minimal signal-dependent noise of optimum control (111). A crisis of thermodynamic entropy is brought about by the breakdown of information flow within the motor system.

## Conclusions

PD predisposes to negative energy balance across its course. While impaired nutritional intake contributes, there are abnormalities on the energy output side. These relate to core motor aspects of the disease, for which the dopaminergic deficit is responsible. The elusive character of bradykinesia is best explained as an under-investment of energy of movement. But when activities are standardised for work performed, PD patients paradoxically expend more energy, suggesting that these inappropriate energy thrift settings are ultimately wasteful.

To subtract energy output from input is to consider energy balance in PD as one might for an engine. This highlights important differences from living things, with their improbable defiance of the second law of thermodynamics. They resist its dispersive effects by managing transformations of their energy. Entropy measurements disclose nothing about the coding scheme or content of basal ganglia neural signals. These information flows are controlling energy exchange through muscular work. Increased neuronal entropy in PD, which coincides with hypokinesia, can be seen as a marker of defective energy transfer.

These enquiries into thermodynamic aspects of PD align with a unifying hypothesis of dopamine's neurotransmitter actions (8)—to adapt energy expenditure to prevailing economic circumstances by influencing the traffic of information about energy.

## Author Contributions

PK and LP-D contributed to the conception, writing and editing of this manuscript, and do agree to be accountable for the content of the work. All authors contributed to the article and approved the submitted version.

## Conflict of Interest

The authors declare that the research was conducted in the absence of any commercial or financial relationships that could be construed as a potential conflict of interest.

## Publisher's Note

All claims expressed in this article are solely those of the authors and do not necessarily represent those of their affiliated organizations, or those of the publisher, the editors and the reviewers. Any product that may be evaluated in this article, or claim that may be made by its manufacturer, is not guaranteed or endorsed by the publisher.
